# Muscle-specific MRI grading of soft tissue involvement provides additional prognostic value beyond skull base criteria in nasopharyngeal carcinoma: a retrospective study

**DOI:** 10.1186/s12885-025-15208-3

**Published:** 2025-11-12

**Authors:** Ping Yang, Sha Liu, Xinghua Chen, Huang Xin, Ying Kong, Ding Liang, Weimin Chen, Tianyu Wu, Ping Zhou, Min Kang

**Affiliations:** 1 Department of Radiation Oncology, The First Affiliated Hospital of Guangxi Medical University, Nanning, Guangxi 530021 China; 2 Department II of Radiotherapy, The First Affiliated Hospital, The First Clinical College of Hainan Medical University, Haikou, Hainan 570100 China; 3 Guangxi Key Laboratory of Early Prevention and Treatment for Regional High Frequency Tumor, Nanning, Guangxi 530021 China; 4 Key Laboratory of Early Prevention and Treatment for Regional High Frequency Tumor, (Guangxi Medical University), Ministry of Education, Nanning, Guangxi 530021 China; 5 Guangxi Key Laboratory of Immunology and Metabolism for Liver Diseases, Nanning, Guangxi 530021 China

**Keywords:** Nasopharyngeal carcinoma, MRI, Soft tissue involvement, Muscle-specific grading, Skull base invasion, Prognosis

## Abstract

**Background:**

In the 9th edition of the AJCC/UICC staging system for nasopharyngeal carcinoma (NPC), soft tissue extension to, but not beyond, the lateral pterygoid (LP) muscle is classified as stage T2 disease. Invasion beyond the LP into the masticator space or infratemporal fossa is designated as T4, while any skull base bone erosion is assigned to T3. However, this framework may oversimplify the prognostic spectrum of soft tissue involvement (STI). This study was formulated to investigate whether a muscle-specific magnetic resonance imaging (MRI) grading system offers incremental prognostic value over current skull base-based staging criteria.

**Methods:**

Patients with newly diagnosed NPC treated with definitive intensity-modulated radiotherapy (IMRT) between 2014 and 2019 were retrospectively analyzed. Pretreatment MRIs were used to categorize STI severity as mild (tensor or levator veli palatini), moderate (prevertebral muscles), or severe (medial/lateral pterygoid or infratemporal fossa). Skull base invasion was classified as either limited (LSBI) or extensive (ESBI). Survival endpoints included local failure-free survival (LFFS), distant metastasis-free survival (DMFS), progression-free survival (PFS), and overall survival (OS). Kaplan–Meier analysis and log-rank tests assessed survival, and Cox proportional hazards models identified independent prognostic factors.

**Results:**

Of 391 patients (median follow-up 88 months; interquartile range, 68–105), 42.9% exhibited mild, 19.4% moderate, and 37.6% severe STI. Five-year survival rates were 84.1% (LFFS), 90.0% (DMFS), 81.3% (PFS), and 80.3% (OS). Survival declined in a stepwise fashion with increasing STI severity (log-rank *P* ≤ 0.0001 for all endpoints); the 5-year OS was 92.9% for mild, 73.7% for moderate, and 68.0% for severe invasion. On multivariable analysis, moderate and severe STI were associated with a 3- to 4-fold increased risk of adverse outcomes, including disease progression and mortality (severe vs. mild OS: HR 4.55, 95% CI 2.47–8.37, *P* < 0.001). Induction chemotherapy was independently protective, reducing the hazard of death by approximately 45% (OS: HR 0.56, 95% CI 0.32–0.97, *P* = 0.04). In contrast, skull base invasion status was not prognostically significant in either univariate or multivariate models. When directly compared, OS for moderate STI and LSBI was similar (81% vs. 78%, *P* = 0.98), while severe STI showed a non-significant trend toward poorer OS compared with LSBI (68.0% vs. 76.1%, *P* = 0.24).

**Conclusions:**

Muscle-specific MRI grading of STI serves as a more robust predictor of treatment outcomes than conventional skull base bone invasion in NPC patients receiving IMRT. This grading system demonstrates potential for refined risk stratification, although prospective, multi-institutional validation is required before clinical implementation.

**Supplementary Information:**

The online version contains supplementary material available at 10.1186/s12885-025-15208-3.

## Introduction

 Nasopharyngeal carcinoma (NPC) is a malignancy with a distinct geographic distribution, particularly prevalent in southern China, Southeast Asia, and North Africa, where it remains a leading cancer type and public health concern. In 2020 alone, NPC accounted for approximately 133,000 new cases and over 80,000 deaths worldwide [1, 2]. Despite improvements in locoregional control through intensity-modulated radiotherapy (IMRT) and advancements in systemic therapy, recurrence, either locally or at distant sites, continues to affect a significant subset of patients [3, 4]. NPC exhibits unique etiological and epidemiological features. Its pathogenesis is multifactorial, involving Epstein-Barr virus (EBV) infection, genetic predisposition, and environmental exposures. These factors contribute to the high incidence of the disease in endemic regions and underscore the need for precise prognostic tools to guide treatment.

The American Joint Committee on Cancer/Union for International Cancer Control (AJCC/UICC) tumor-node-metastasis (TNM) staging system is the global standard for clinical decision-making in NPC. In its 9th edition, soft tissue involvement (STI) is categorized broadly: tumor extension to the parapharyngeal space or adjacent muscular structures such as the tensor veli palatini (TVP), levator veli palatini (LVP), prevertebral, or medial/lateral pterygoid (MP, LP) muscles is classified as T2 disease, while extension beyond the outer border of the LP into the masticator space or infratemporal fossa is classified as T4. Any skull base bone involvement is designated T3, regardless of its anatomical extent or aggressiveness. However, the current staging system does not distinguish between different muscles within the T2 category, treating all STI equally as T2 disease [5].

However, emerging MRI-based evidence challenges this simplified classification. Studies have shown that midline skull base erosion (e.g., of the clivus or pterygoid process) is associated with a better prognosis than lateral invasion involving neural foramina [6, 7]. Similarly, limited muscular involvement such as that affecting the TVP or LVP muscles may be indicative of more favorable outcomes compared to tumor extension into the prevertebral region or deep muscle compartments like the LP or MP [8–11]. Previous investigations have used various methods to assess soft tissue invasion, Zhang et al. employed a binary classification system for distinguishing between parapharyngeal from masticator space involvement linking poor prognosis with lateral extension [12]. Dong et al. utilized network-based approaches to analyze STI patterns [13], while Cheng et al. focused primarily on skull base invasion grading [14]. However, these studies have largely treated soft tissue spread as either binary variables or have not systematically evaluated the prognostic significance of sequential muscle invasion patterns following anatomical pathways.

In contrast to these earlier approaches, the present study introduces a novel sequential, depth-based muscle-specific grading system that systematically follows anatomical invasion pathways from superficial (TVP/LVP) to intermediate (prevertebral) to deep (pterygoid/infratemporal) muscular structures. This grading system is designed as an adjunct risk stratification tool rather than a replacement for existing TNM staging. It aims to provide granular prognostic information within the current T2 category, potentially informing decisions regarding treatment intensification without disruption of the established TNM framework.

Magnetic resonance imaging (MRI) has become the cornerstone for local staging in NPC due to its superior soft tissue resolution and sensitivity in detecting perineural spread, muscular infiltration, and skull base invasion. MRI facilitates accurate mapping of tumor spread in anatomically complex regions, such as the parapharyngeal and masticator spaces. The complex muscular architecture surrounding the nasopharynx, including the TVP, LVP, prevertebral muscles, and the MP and LP muscles, offers a series of distinct potential pathways for tumor dissemination. Identifying specific patterns of muscle involvement following these natural anatomical progression routes is essential for optimizing treatment strategies and improving prognostication.

Accordingly, the present study was developed with the following aims: (1) to assess whether a sequential, muscle-specific grading of STI confers independent prognostic value in NPC; (2) to evaluate the prognostic utility of this depth-based grading system compared to conventional skull base criteria; and (3) to demonstrate the potential clinical utility of this approach as a complementary risk stratification tool that could inform future T2 sub-classifications (e.g., T2a, T2b, T2c) if validated across multiple institutions.

## Materials and methods

### Participants

This retrospective study analyzed 391 consecutive patients newly diagnosed with NPC at the Department of Radiotherapy of the First Affiliated Hospital of Guangxi Medical University, between January 2014 and December 2019. Inclusion criteria were: (1) histologically confirmed, treatment-naïve NPC; (2) availability of comprehensive clinical and laboratory data at diagnosis; (3) high-resolution pre-treatment MRI of the nasopharynx and cervical region; (4) no prior history of antineoplastic therapy; and (5) completion of radical IMRT. Patients were excluded if they: (1) were lost to follow-up during treatment or subsequent monitoring; (2) were diagnosed with synchronous malignancies; (3) developed second primary cancers post-treatment; (4) were pregnant or lactating; or (5) were medically unfit to complete treatment. These criteria were chosen to minimize bias and ensure consistency in outcome assessment. All patients underwent a standardized pretreatment evaluation protocol, including medical history, physical examination, hematologic and biochemical profiling, nasopharyngoscopy, contrast-enhanced MRI of the head and neck, chest radiography, abdominal ultrasound, and skeletal assessment via whole-body bone scintigraphy or 18 F-FDG PET/CT, depending on clinical indications. Plasma EBV-DNA levels were quantified via real-time PCR, in accordance with previously validated protocols [7, 12–14].This study was conducted in compliance with the Declaration of Helsinki and approved by the Ethics Committee of the First Affiliated Hospital of Guangxi Medical University. Given its retrospective nature, the requirement for informed consent was waived. Patient confidentiality and data anonymity were maintained throughout. To reduce selection bias, all eligible patients within the study period who met inclusion criteria were enrolled without exception.

### Imaging assessments

All patients underwent pre-treatment MRI using a 1.5-T GE Signa scanner. The imaging protocol incorporated both conventional and contrast-enhanced spin-echo sequences, including T2-weighted imaging (T2WI; TR = 3000–4000 ms, TE = 102–110 ms), T1-weighted imaging (T1WI; TR = 2200–2400 ms, TE = 77–109 ms, T1 = 750 ms), and contrast-enhanced T1WI following intravenous administration of 15 mL of gadolinium-diethylenetriaminepentaacetic acid (Gd-DTPA). Scans were performed in axial, sagittal, and coronal planes using a head coil, with a slice thickness of 6 mm and an interslice gap of 1 mm (matrix: 256 × 192), covering the area from the suprasellar cistern to the inferior clavicular margin. This protocol was optimized to detect soft tissue extension and skull base involvement, with particular focus on delineating the boundaries of the parapharyngeal and masticator spaces.

Two board-certified radiologists independently reviewed all MRI scans while blinded to patient outcomes. To distinguish true tumor invasion from secondary muscle inflammation, the following MRI criteria were applied: (1) signal characteristics showing intermediate to high signal intensity on T2WI with enhancement patterns similar to the primary tumor on contrast-enhanced T1WI; (2) morphological features, including focal, asymmetric muscle enlargement with loss of the normal muscle architecture; (3) heterogeneous enhancement patterns distinct from the more homogeneous enhancement typically seen with inflammatory changes; and (4) anatomical continuity with the primary nasopharyngeal tumor. Discrepancies were resolved by consensus, and the final adjudicated (consensus) grade was used for analysis. Because per-case adjudication logs were not systematically archived in early years, exact counts of consensus cases are unavailable; quantitative inter-observer agreement measures were not performed.

**Fig. 1:**
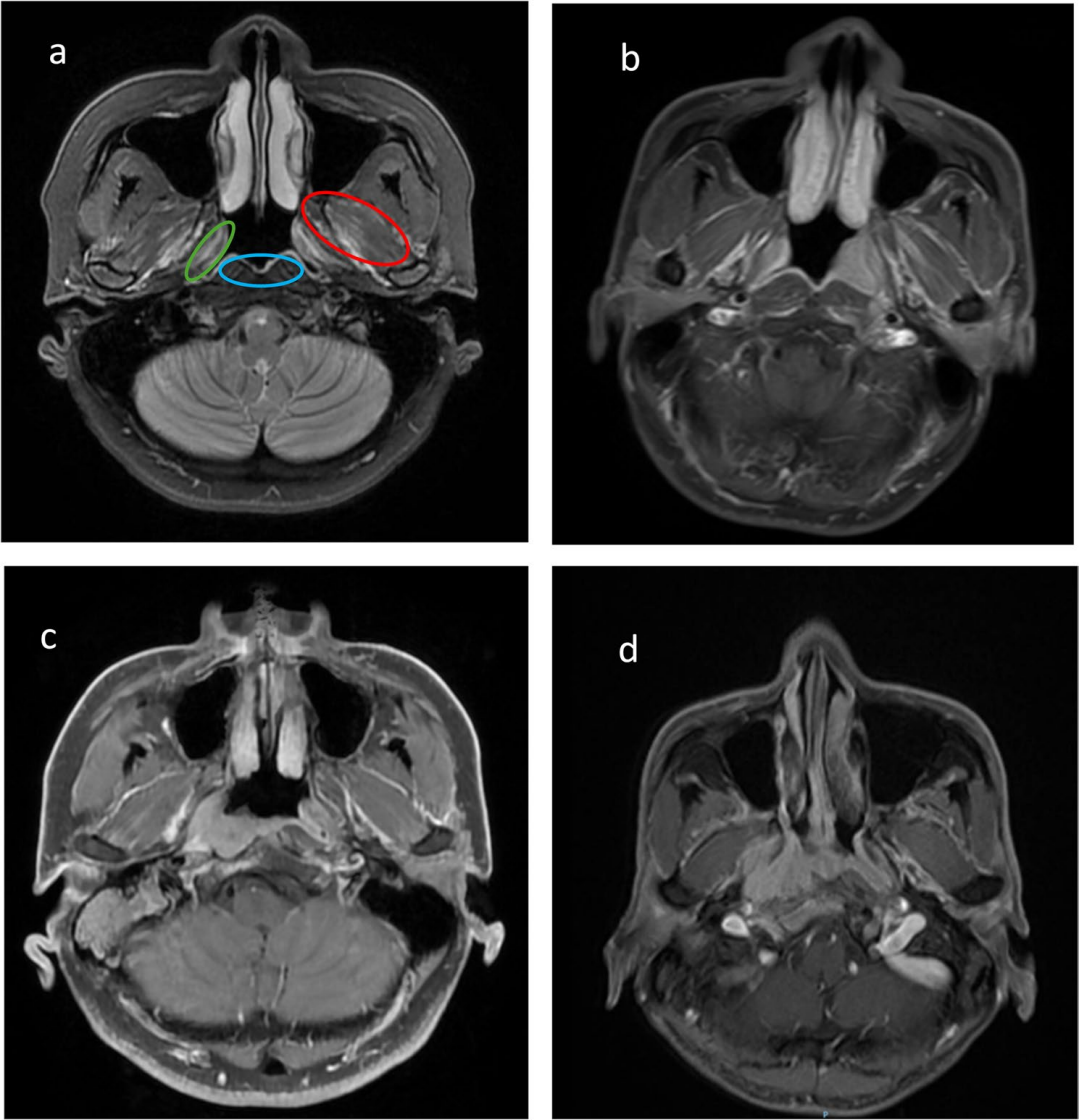
Representative magnetic resonance images (MRI) for different STI grades. **a** Axial T2-weighted MR image of normal nasopharynx displays the distribution area of STI. If the tumor invades the green, blue and red areas, they represent mild, moderate, and severe soft tissue invasion, respectively. **b** Contrast-enhanced T1-weighted images in a 42-year-old man. The nasopharynx lesions are accompanied only by LVP invasion (arrow), which is defined as mild STI. **c** Contrast-enhanced T1-weighted mages in a 48-year-old man with nasopharynx lesions extending to the prevertebral muscle (arrow), which is defined as moderate STI. **d** Contrast-enhanced T1-weighted images in a 56-year-old woman show the extensive invasion of LP (arrow), which is defined as severe STI. STI, soft tissue involvement; LVP, levator veli palatini; LP, lateral pterygoid

Based on the deepest anatomical site of muscle infiltration, STI was categorized into three muscle-specific grades: mild (TVP or LVP muscles), moderate (prevertebral muscles), and severe (MP or LP muscles and/or infratemporal fossa involvement) (Fig. 1). In parallel, skull base invasion was stratified as limited or extensive, following previously established imaging criteria [7, 14]. This grading system was guided by both anatomical insights and prior literature suggesting that the depth of muscle invasion correlates with clinical outcomes.

### Treatment protocol

All patients received definitive IMRT according to a uniform protocol consistent with guidelines from the International Commission on Radiation Units and Measurements (ICRU) Reports 50 and 62 [15, 16]. Radiotherapy target volumes included: the primary nasopharyngeal tumor (GTV nx), gross nodal disease (GTV nd), the surrounding high-risk subclinical zone (CTV1), and the elective cervical lymphatic drainage regions (CTV2). Prescribed radiation doses were as follows: GTV nx: 68–76 Gy in 30–33 fractions; GTV nd: 66–70 Gy in 30–33 fractions; CTV1: 60–64 Gy in 30–33 fractions; CTV2: 50–54 Gy in 30–33 fractions. Daily fraction sizes ranged from 2.00 to 2.33 Gy. Full technical details are available in earlier reports [17–20]. Tumors were restaged according to the 9th edition AJCC system [5].

Most patients received induction chemotherapy (IC) followed by concurrent chemoradiotherapy (CCRT), with or without adjuvant chemotherapy (AC). IC regimens were administered every 21 days and included one of the following: TP: docetaxel 75 mg/m² + cisplatin 75 mg/m², both on day 1; PF: cisplatin 80 mg/m² (day 1) + 5-fluorouracil 800–1000 mg/m² (days 1–5, continuous infusion); TPF: docetaxel 60 mg/m² + cisplatin 60 mg/m² (day 1), with 5-fluorouracil 600 mg/m² (days 1–5, continuous infusion). While undergoing radiotherapy, concurrent cisplatin was given either weekly or triweekly. In patients unable to tolerate or deemed ineligible for cisplatin, alternative platinum agents were used. AC regimens were identical to those used during IC.

### Clinical endpoints and follow up

Primary clinical endpoints included: local failure-free survival (LFFS), measured time from diagnosis to first occurrence of local recurrence/persistence or censoring at last follow-up/death; distant metastasis-free survival (DMFS), measured as the time to first detection of distant metastasis or censoring; progression-free survival (PFS), measured as the time to any progression (local, regional, or distant), death, or censoring; and overall survival (OS), measured as the time to death from any cause or censoring. Patients were monitored post-treatment every three months for the first two years, then every six months up to five years, or until death. Each follow-up included MRI of the nasopharynx and neck, and CT imaging of the chest and abdomen. Where recurrence or metastasis was suspected, diagnostic confirmation was obtained via fine needle aspiration or histopathologic biopsy when necessary. Patients lost to follow-up or alive without recurrence at study end were censored at their most recent assessment.

### Statistical analysis

Continuous variables were expressed as medians with interquartile ranges (IQR) and compared across muscle invasion categories using one-way ANOVA or the Kruskal–Wallis test, depending on distribution. Categorical data were summarized as frequencies (percentages) and compared using χ² or Fisher’s exact tests. Kaplan–Meier survival curves were constructed for LFFS, DMFS, PFS, and OS, with differences assessed via log-rank testing. Univariable analyses identified candidate prognostic variables, which were subsequently entered into multivariable Cox proportional hazards models to compute adjusted hazard ratios (HRs) and 95% confidence intervals (CIs). Statistical analyses were conducted using SPSS 26.0 (IBM Corp) and R v2025.05.1 + 513(R Foundation for Statistical Computing). A two-sided *P* < 0.05 was deemed significant.

## Results

### Patient characteristics

Baseline characteristics are detailed in Table 1. The cohort included 391 patients, classified by MRI muscle invasion grading into mild (*n* = 168, 42.9%), moderate (*n* = 76, 19.4%), and severe (*n* = 147, 37.6%) groups. The median age was 46 years (IQR: 37–53), and sex distribution was predominantly male (74.4%), with no significant differences in age or sex across the three groups (all *P* > 0.05). Alcohol consumption varied significantly, with the highest prevalence in the moderate group (47.4%) and the lowest in the mild group (30.4%; *P* = 0.037). Smoking status did not differ statistically (*P* = 0.067). Disease severity increased with muscle invasion depth: T4 classification was observed in 54.6% of severe cases, 28.4% of moderate cases, and 11.0% of mild cases (*P* < 0.001). Similarly, stage IV disease was more prevalent in the severe (61.0%) compared to the moderate (41.8%) and mild (34.4%) groups (*P* < 0.001). N-category distributions were not significantly different (*P* = 0.20). EBV-DNA positivity showed a heterogeneous distribution, with 58.5% in mild, 31.0% in moderate, and 71.7% in severe groups (*P* = 0.007), though interpretation is limited by substantial missing data (64.2%). Extensive skull base invasion (ESBI) was most common in the severe group (62.2%), followed by moderate (48.7%) and mild (40.8%) (*P* = 0.003). Treatment regimens were well balanced across groups: 85.7% received IC, 92.8% underwent CCRT, and 34.5% received AC, with no significant intergroup differences (all *P* > 0.10). Among 391 patients, 335 (85.7%) received IC; among these, TP accounted for 280 (83.6%), PF 30 (9.0%), and TPF 25 (7.5%), while 56 (14.3%) of the cohort received no IC. Baseline EBV DNA was available in 140/391 (35.8%) patients; among those tested, 80 (57.1%) were positive and 60 (42.9%) were negative. Using the total cohort as the denominator, this corresponds to 80/391 (20.5%) positive, 60/391 (15.3%) negative, and 251/391 (64.2%) missing.

**Table 1:** Patient characteristics

Characteristics	Overall *N* = 391^1^	Mild *N* = 168^1^	Moderate *N* = 76^1^	Severe *N* = 147^1^	*P*-value^2^
Sex					0.2
Male	291 (74.4%)	119 (70.8%)	62 (81.6%)	110 (74.8%)	
Female	100 (25.6%)	49 (29.2%)	14 (18.4%)	37 (25.2%)	
Age	45.14 ± 10.63	44.70 ± 10.79	44.42 ± 10.60	46.02 ± 10.49	0.3
Drinking					**0.037**
No	250 (63.9%)	117 (69.6%)	40 (52.6%)	93 (63.3%)	
Yes	141 (36.1%)	51 (30.4%)	36 (47.4%)	54 (36.7%)	
Smoking					0.067
No	231 (59.1%)	110 (65.5%)	39 (51.3%)	82 (55.8%)	
Yes	160 (40.9%)	58 (34.5%)	37 (48.7%)	65 (44.2%)	
Pathology (WHO)					0.5
I-II	31 (7.9%)	16 (9.5%)	4 (5.3%)	11 (7.5%)	
III	360 (92.1%)	152 (90.5%)	72 (94.7%)	136 (92.5%)	
EBV-DNA					**0.007**
Negative	60 (15.3%)	27 (16.1%)	20 (26.3%)	13 (8.8%)	
Positive	80 (20.5%)	38 (22.6%)	9 (11.8%)	33 (22.4%)	
Data missing	251 (64.2%)	103 (61.3%)	47 (61.8%)	101 (68.7%)	
T classification					**< 0.001**
T1	58 (15.6%)	52 (31.9%)	3 (4.5%)	3 (2.1%)	
T2	70 (18.9%)	38 (23.3%)	14 (20.9%)	18 (12.8%)	
T3	129 (34.8%)	55 (33.7%)	31 (46.3%)	43 (30.5%)	
T4	114 (30.7%)	18 (11.0%)	19 (28.4%)	77 (54.6%)	
N classification					0.2
N0	18 (4.9%)	7 (4.3%)	3 (4.5%)	8 (5.7%)	
N1	69 (18.6%)	33 (20.2%)	12 (17.9%)	24 (17.0%)	
N2	221 (59.6%)	86 (52.8%)	44 (65.7%)	91 (64.5%)	
N3	63 (17.0%)	37 (22.7%)	8 (11.9%)	18 (12.8%)	
Overall Stage (9th)					**< 0.001**
1–2	34 (9.2%)	22 (13.5%)	6 (9.0%)	6 (4.3%)	
3	167 (45.0%)	85 (52.1%)	33 (49.3%)	49 (34.8%)	
4	170 (45.8%)	56 (34.4%)	28 (41.8%)	86 (61.0%)	
Skull-base invasion					**0.003**
ESBI	122 (51.0%)	29 (40.8%)	19 (38.8%)	74 (62.2%)	
LSBI	117 (49.0%)	42 (59.2%)	30 (61.2%)	45 (37.8%)	
Induction chemotherapy					0.10
No	55 (14.1%)	18 (10.7%)	16 (21.1%)	21 (14.3%)	
Yes	336 (85.9%)	150 (89.3%)	60 (78.9%)	126 (85.7%)	
Concurrent chemotherapy					0.7
No	28 (7.2%)	14 (8.3%)	4 (5.3%)	10 (6.8%)	
Yes	363 (92.8%)	154 (91.7%)	72 (94.7%)	137 (93.2%)	
Adjuvant chemotherapy					0.3
No	256 (65.5%)	106 (63.1%)	47 (61.8%)	103 (70.1%)	
Yes	135 (34.5%)	62 (36.9%)	29 (38.2%)	44 (29.9%)	

Bold indicates a significant difference among groups with *p* < 0.05

*Abbreviations:* *EBV* Epstein-Barr virus, *ESBI* extensive skull-base invasion, *LSBI* limited skull-base invasion

^1^n (%); Mean ± SD; ^2^Pearson’s Chi-squared test; Kruskal-Wallisr ank sum test

### Survival outcomes by depth of invasion

Unlike traditional staging parameters (Supplementary Figures S1-S2), the muscle-specific grading system described here enabled significant prognostic stratification across all endpoints. Kaplan–Meier survival analysis revealed significant differences across the three muscle invasion grades for all four survival outcomes.

**Fig. 2:**
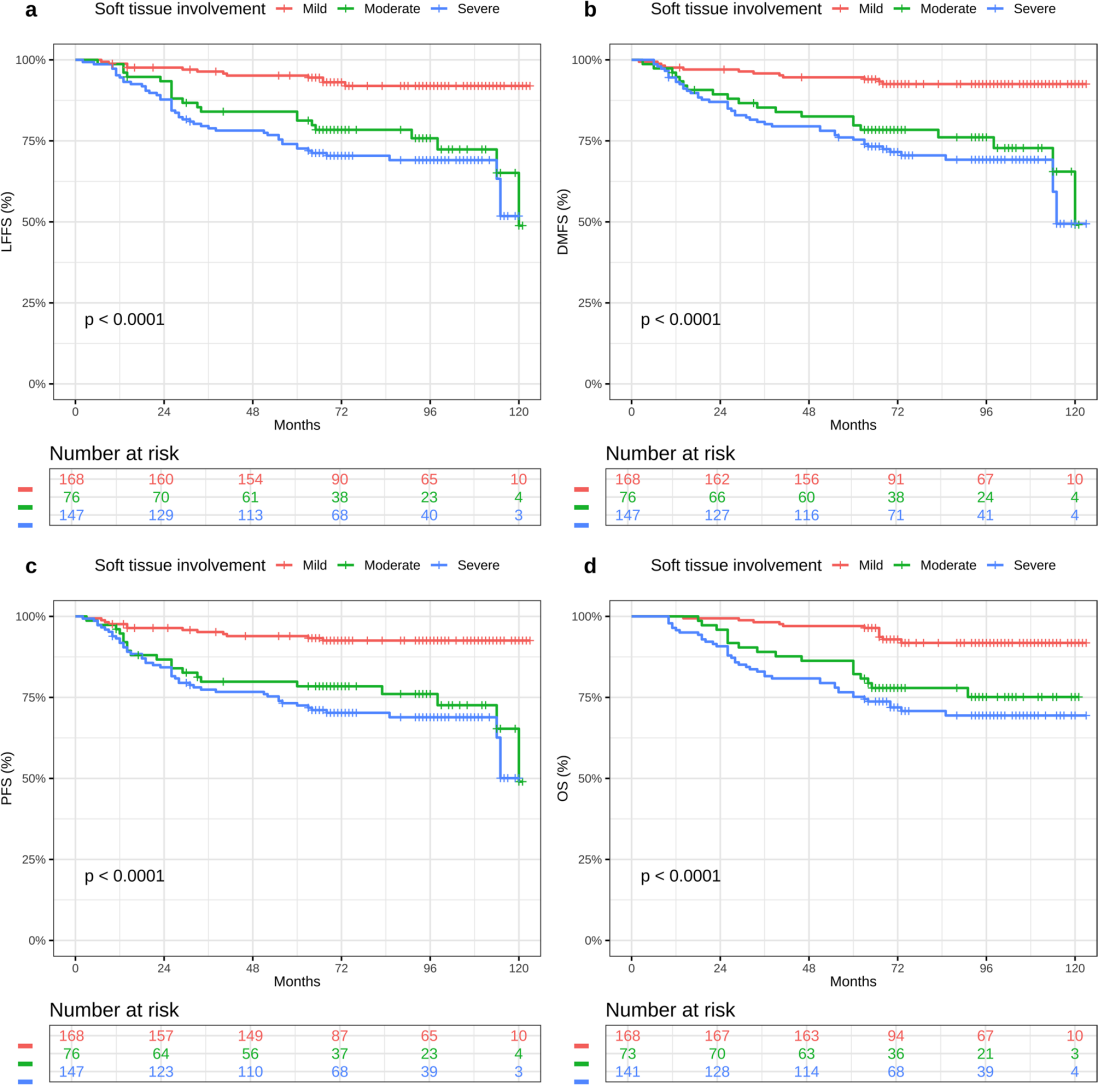
Kaplan-Meier survival curves of (LFFS) **a**, (DMFS) **b**, (PFS) **c** and (OS) **d** in mild, moderate, and severe STI. Abbreviations: LFFS, local failure-free survival; DMFS, distant metastasis-free survival; PFS, progression-free survival; OS, overall survival rates; STI, soft tissue involvement

**Fig. 3:**
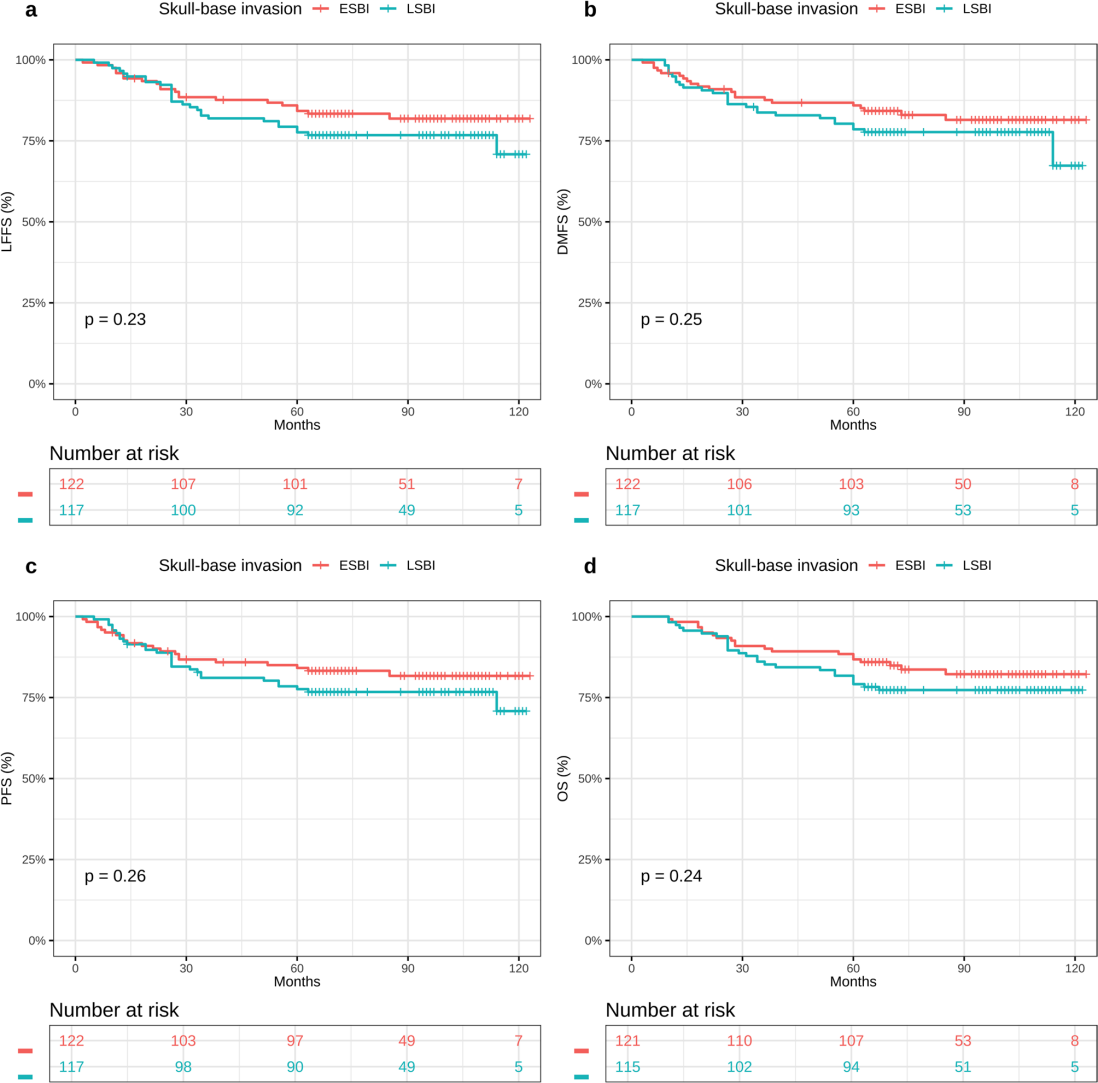
Kaplan-Meier survival curves of (LFFS) **a** (DMFS) **b** (PFS) **c** and (OS) **d** in ESBI and LSBI. Abbreviations: LFFS, local failure-free survival; DMFS, distant metastasis-free survival PFS, progression-free survival; OS, overall survival rates; ESBI, extensive skull-base invasion; LSBI, limited skull-base invasion

At a median follow-up of 88 months (IQR: 68–105), 73 of the 391 patients (18.7%) experienced treatment failure (11.9% vs. 26.3% vs. 22.4%, *P* = 0.009). The details of the treatment failure are listed in Table 2. Rates of locoregional failure varied significantly by muscle invasion grade: 11.3% (19/168) in the mild group, 23.7% (18/76) in the moderate group, and 17.0% (25/147) in the severe group (*P* = 0.044). Similarly, the incidence of distant metastasis rose with increasing invasion severity: 4.8% in mild, 15.8% in moderate, and 12.9% in severe categories (*P* = 0.009). When stratified by skull-base invasion, there was no significant difference in overall treatment failure between limited and extensive involvement (16.2% vs. 18.9%, respectively; *P* = 0.718). The estimated 5-year survival outcomes for the full cohort were as follows: LFFS 84.1%, DMFS 90.0%, PFS 81.3%, and OS 80.3%. When stratifying by muscle-invasion depth, 5-year LFFS rates were 88.7% (mild), 76.3% (moderate), and 83.0% (severe) (*P* = 0.0001), with corresponding DMFS rates of 95.2%, 84.2%, and 87.1% (*P* < 0.0001), PFS rates of 88.1%, 73.7%, and 77.6% (*P* < 0.0001), and OS rates of 92.9%, 73.7%, and 68.0% (*P* < 0.0001) (Fig. 2). In contrast, patients grouped by skull-base invasion (limited vs. extensive) exhibited no significant differences in survival outcomes for LFFS (88.0% vs. 83.6%; *P* = 0.23), DMFS (91.5% vs. 89.3%; *P* = 0.25), PFS (83.8% vs. 81.1%; *P* = 0.26), or OS (76.1% vs. 82.8%; *P* = 0.24) (Fig. 3).

**Table 2:** Patterns of treatment failure for patients with soft tissue involvement after IMRT

Treatment Failure Pattern	Mild *N* = 168^1^	Moderate *N* = 76^1^	Severe *N* = 147^1^	*P* ^2^
Distant only	1 (0.6%)	2 (2.6%)	8 (5.4%)	**0.024**
Bone	1 (100.0%)	2 (100.0%)	1 (12.5%)	
Bone and liver	0 (0.0%)	0 (0.0%)	1 (12.5%)	
Bone, lung and liver	0 (0.0%)	0 (0.0%)	1 (12.5%)	
Liver	0 (0.0%)	0 (0.0%)	1 (12.5%)	
Lung	0 (0.0%)	0 (0.0%)	2 (25.0%)	
Other	0 (0.0%)	0 (0.0%)	2 (25.0%)	
Local and distant	6 (3.6%)	9 (11.8%)	11 (7.5%)	**0.049**
Regional and distant	1 (0.6%)	1 (1.3%)	0 (0.0%)	0.5
Local and regional	1 (0.6%)	1 (1.3%)	2 (1.4%)	0.7
Local only	5 (3.0%)	5 (6.6%)	10 (6.8%)	0.2
Regional only	6 (3.6%)	2 (2.6%)	2 (1.4%)	0.5
Total	20 (11.9%)	20 (26.3%)	33 (22.4%)	**0.009**

Bold indicates a significant difference among groups with *p* < 0.05

*Abbreviations:* *IMRT* intensity-modulated radiotherapy

^1^n (%); ^2^Fisher’s exact test; Pearson’s Chi-squared test

### Univariate and multivariate analyses

The prognostic relevance of multiple clinical and treatment-related factors, including age, sex, muscle invasion grade, skull-base involvement, TN staging, EBV-DNA status, and receipt of IC, concurrent chemotherapy, and AC, was next assessed for all four survival endpoints. In univariate analysis, both muscle-specific tumor invasion and receipt of IC were significantly associated with all endpoints. Age showed a marginal association with overall survival (OS; HR 1.02 per year; *P* = 0.056). Other variables such as sex, alcohol or tobacco use, histological subtype, EBV-DNA status, T and N stage, overall clinical stage, skull-base involvement, and concurrent chemotherapy or AC did not attain significance (all *P* > 0.20) (Table 3). Multivariate Cox regression confirmed that both STI grade and use of IC retained independent prognostic value. Compared with mild muscle invasion, moderate involvement significantly increased the risk of locoregional failure by more than three-fold, as reflected by LFFS (HR 3.30), DMFS (HR 3.43), PFS (HR 3.39), and OS (HR 3.39) outcomes (all *P* ≈ 0.001). Severe muscle invasion conferred even higher risks across all endpoints, with hazard ratios between 4.4 and 4.6 (all *P* < 0.001). Conversely, IC treatment independently reduced the likelihood of treatment failure by approximately 45%, as reflected by OS (HR 0.56), PFS (HR 0.56), DMFS (HR 0.57), and LFFS (HR 0.55) outcomes (all *P* ≈ 0.04). While increasing age trended toward worse OS (HR 1.02/year; *P* = 0.074), other covariates, including skull-base status and nodal involvement, were excluded from final models due to lack of significance (Table 4).

**Table 3:** Univariate analysis of variables correlated with various clinical endpoints

Variables	LFFS		DMFS		PFS		OS	
	HR (95% CI)	*P* value	HR (95% CI)	*P* value	HR (95% CI)	*P* value	HR (95% CI)	*P* value
Sex	0.699 (0.39–1.255)	0.231	0.703 (0.392–1.261)	0.237	0.705 (0.393–1.264)	0.240	0.696 (0.388–1.25)	0.225
Age	1.021 (0.999–1.044)	0.067	1.021 (0.999–1.045)	0.064	1.021 (0.999–1.044)	0.067	1.022 (0.999–1.045)	0.056
Drinking	1.089 (0.674–1.759)	0.727	1.048 (0.649–1.692)	0.849	1.06 (0.657–1.712)	0.811	1.072 (0.664–1.732)	0.775
Smoking	1.384 (0.869–2.204)	0.172	1.328 (0.834–2.115)	0.233	1.35 (0.848–2.151)	0.206	1.354 (0.85–2.157)	0.202
Pathology (WHO)	1.549 (0.565–4.247)	0.395	1.499 (0.547–4.112)	0.431	1.528 (0.557–4.189)	0.410	1.5 (0.547–4.113)	0.431
EBV-DNA	1.187 (0.543–2.591)	0.668	1.203 (0.551–2.627)	0.643	1.161 (0.531–2.535)	0.709	1.221 (0.559–2.667)	0.616
T classification	1.267 (0.518–3.099)	0.605	1.261 (0.515–3.085)	0.611	1.291 (0.528–3.158)	0.576	1.21 (0.494–2.96)	0.677
N classification	0.536 (0.161–1.781)	0.309	0.538 (0.162–1.786)	0.311	0.524 (0.158–1.741)	0.291	0.552 (0.166–1.832)	0.332
Overall Stage (9th)	1.107 (0.427–2.866)	0.835	1.133 (0.438–2.935)	0.797	1.12 (0.433–2.901)	0.815	1.109 (0.428–2.871)	0.832
Soft tissue invasion	3.553 (1.697–7.44)	0.001**	3.636 (1.736–7.615)	0.001**	3.618 (1.727–7.576)	0.001**	3.603 (1.72–7.545)	0.001**
Skull-base invasion	1.399 (0.781–2.506)	0.259	1.402 (0.783–2.512)	0.256	1.378 (0.769–2.469)	0.281	1.421 (0.793–2.546)	0.237
Induction chemotherapy	0.525 (0.3–0.919.3.919)	0.024*	0.507 (0.289–0.889)	0.018*	0.517 (0.296–0.904)	0.021*	0.515 (0.294–0.904)	0.021*
Concurrent chemotherapy	1.013 (0.408–2.515)	0.978	1.004 (0.404–2.492)	0.993	1.017 (0.41–2.525)	0.971	0.976 (0.393–2.423)	0.958
Adjuvant chemotherapy	1.326 (0.826–2.129)	0.243	1.339 (0.834–2.149)	0.228	1.318 (0.821–2.115)	0.254	1.327 (0.826–2.13)	0.242

Bold indicates statistically significant with *p* < 0.05

*Abbreviations:* *HR* hazard ratio, *CI* confidence interval, *LFFS* locoregional recurrence-free survival, *DMFS* distant metastasis-free survival, *PFS* progression-free survival, *OS* overall survival

*P < 0.05; **P < 0.01

**Table 4:** Multivariate analysis of variables correlated with various clinical endpoints

Variables	LFFS		DMFS		PFS		OS	
	HR (95% CI)	*P* value	HR (95% CI)	*P* value	HR (95% CI)	*P* value	HR (95% CI)	*P* value
Soft tissue involvement (moderate vs. mild)	3.305 (1.571–6.953)	0.002	3.429 (1.632–7.204)	0.001	3.385 (1.609–7.119)	0.001	3.387 (1.612–7.119)	0.001
Soft tissue involvement (severe vs. mild)	4.491 (2.359–8.55)	**< 0.001**	4.409 (2.315–8.4.315.4)	**< 0.001**	4.367 (2.293–8.317)	**< 0.001**	4.605 (2.418–8.769)	**< 0.001**
Induction chemotherapy (yes vs. no)	0.551 (0.313–0.971)	**0.039**	0.567 (0.322–0.996)	**0.048**	0.56 (0.319–0.985)	**0.044**	0.562 (0.319–0.989)	**0.046**
Age (per year increase)	1.02 (0.997–1.044)	0.082	1.02 (0.997–1.043)	0.091	1.02 (0.997–1.043)	0.087	1.021 (0.998–1.045)	0.074

Bold indicates statistically significant with *p* < 0.05

*Abbreviations:* *HR* hazard ratio, *CI* confidence interval, *LFFS* locoregional recurrence-free survival, *DMFS* distant metastasis-free survival, *PFS* progression-free survival, *OS* overall survival

### Comparative analysis of anatomical classifications

**Fig. 4:**
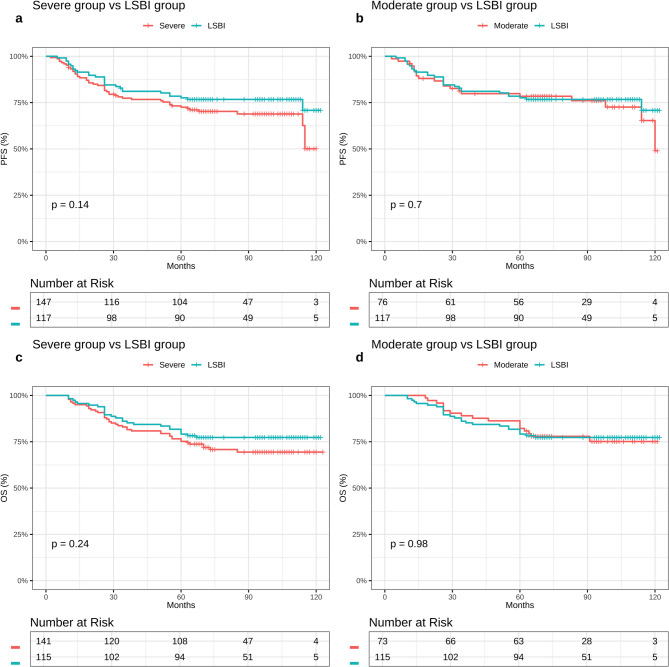
Kaplan-Meier survival curves of PFS **a**, **b** and OS **c**, **d** in severe STI group vs LSBI group; moderate STI group vs LSBI group. Abbreviations: PFS, progression-free survival; OS, overall survival rates; STI, soft tissue involvement; LSBI, limited skull-base invasion

To assess the relative prognostic value of bone versus muscle invasion, survival outcomes were compared between patients with LSBI and those with severe muscle infiltration. The LSBI group achieved 5-year PFS and OS rates of 83.8% and 76.1%, respectively, which were not significantly different from the severe muscle-invasion cohort (77.6% and 68.0%; P = 0.14 and 0.24). Moreover, LSBI outcomes closely resembled those of the moderate muscle-invasion group (PFS 84% vs. 86%, P = 0.70; OS 78% vs. 81%, P = 0.98; Fig. 4).

## Discussion

This study highlights the prognostic utility of a muscle-specific MRI-based grading system for stratifying NPC, revealing survival differences not captured by conventional classifications of skull-base invasion. In this cohort, 5-year LFFS, DMFS, PFS, and OS showed a clear stepwise decline with increasing depth of muscular invasion. Severe muscle involvement emerged as an independent predictor of poorer outcomes, associated with nearly a threefold higher risk of mortality (HR ≈ 2.9, 95% CI 1.7–4.8), even after adjusting for TNM stage, EBV-DNA levels, and treatment factors. Conversely, the extent of skull-base invasion did not significantly influence any evaluated survival outcome. MRI is currently the preferred modality for NPC staging due to its superior soft-tissue resolution and enhanced sensitivity for detecting skull-base and cranial nerve involvement [21, 22]. Its ability to detect subtle signal alterations associated with early STI makes it particularly effective in identifying tumor spread patterns [23–25]. The distinct prognostic gradient observed in the present study is consistent with the biological rationale that increasing depth of muscle infiltration is inversely correlated with prognosis in T2 NPC [13, 26]. One explanation is that mild invasion is typically associated with smaller tumors that encroach only minimally on peri-nasopharyngeal tissue, leading to lower tumor burden and improved local control. In contrast, extensive muscle involvement often coincides with larger primary lesions capable of penetrating deep fascial planes and exploiting perineural routes through skull-base foramina, thereby increasing the risk of early systemic dissemination and distant metastasis. Unlike the classification used by Zhang et al., which broadly categorized invasion into parapharyngeal or masticator space and linked poor prognosis with lateral extension, most prior studies have not specifically assessed individual muscle involvement [12]. The present findings build upon this foundation by demonstrating a sequential decline in survival outcomes corresponding to invasion of the palatal, prevertebral, and pterygoid muscles, reinforcing the notion that deeper or more lateral STI is associated with adverse prognosis [27, 28]. Several MRI-based studies, including those by Cheng et al. and Li et al., have highlighted the prognostic significance of skull-base bone invasion, reporting worse outcomes in cases of extensive involvement [14, 29]. However, in the present study cohort, no significant differences in 5-year LFFS, DMFS, PFS, or OS were observed between patients with LSBI vs. ESBI (all *p* >0.20). Furthermore, skull-base status did not emerge as a prognostic factor in either univariate or multivariable analysis. This divergence from previous reports may be attributed to several factors. First, MRI-detected skull-base invasion often represents non-measurable or semi-quantitative findings, with the classification of limited versus extensive involvement being highly subjective and dependent on radiologists’ interpretation of marrow or cortical signal alterations. Second, modern high-resolution MRI is capable of detecting subtle changes such as edema or cortical thinning, which may lead to overestimation of disease extent and reduce the clinical utility of this subclassification. Finally, the implementation of IMRT, which provides precise and uniform dose coverage across the skull base, likely mitigates the clinical impact of bone involvement, thereby equalizing outcomes regardless of invasion extent.

The unfavorable prognosis linked to deep muscle invasion in NPC arises from a complex interplay of biological and anatomical factors. Tumor infiltration into the prevertebral and pterygoid musculature likely reflects a more aggressive tumor phenotype, associated with increased proliferative activity, heightened invasiveness, and greater potential for perineural and hematogenous dissemination. At the molecular level, tumors exhibiting extensive STI frequently show elevated expression of matrix metalloproteinases, markers of epithelial–mesenchymal transition, and pro-angiogenic mediators. These molecular alterations promote degradation of the extracellular matrix, compromise anatomical barriers, and facilitate early spread to lymphatic and distant sites. Moreover, the anatomical proximity of the pterygoid region and infratemporal fossa to critical neurovascular structures increases the likelihood of cranial nerve encroachment and may hinder surgical salvage options in recurrent disease. The anatomical intricacy of these areas also presents challenges to achieving complete resection, potentially explaining the elevated rates of local failure observed in patients with deep muscle involvement. Additionally, the dense vascular and lymphatic architecture of deep muscular compartments offers multiple conduits for systemic dissemination, aligning with the increased incidence of distant metastases seen in this subgroup.

Integrating a muscle-specific MRI-based grading system into routine NPC staging could significantly enhance prognostic accuracy compared to the current AJCC/UICC 9th-edition framework, which collectively classifies all muscular involvement as T2 disease. This aggregated classification may obscure clinically relevant heterogeneity. Patients with severe muscle involvement represent a high-risk subgroup that may warrant closer monitoring and consideration for investigational approaches, while those with mild invasion demonstrate more favorable outcomes and could be studied in future trials examining optimized treatment approaches. Standardizing radiologic assessments to routinely document specific patterns of muscle involvement would facilitate more consistent interdisciplinary communication. Such standardization could support personalized surveillance protocols, such as more frequent MRI for patients at elevated risk, and enable the integration of quantitative imaging biomarkers and serial plasma EBV-DNA monitoring into clinical workflows. Multi-center, prospective validation studies and health-economic evaluations are now needed to confirm that this anatomically nuanced approach can improve outcomes while maintaining cost-effectiveness.

The prognostic evaluation of NPC cases will likely increasingly rely on a combination of anatomical imaging, molecular profiling, and functional imaging metrics in the future. Plasma EBV-DNA has already been established as a robust surrogate for tumor burden and therapeutic response. When combined with muscle-specific MRI stratification, radiomic signatures, and gene expression profiling, it may enable the development of highly individualized risk models. These tools could guide treatment decisions by balancing therapeutic intensity with long-term toxicity and quality-of-life considerations. Additionally, advances in liquid biopsy, including circulating tumor DNA and microRNA profiling, offer further opportunities to refine risk stratification. Integration of such molecular data with detailed anatomical grading may yield robust composite prognostic tools, supporting more precise patient selection for emerging therapies.

Validation of the clinical implementation of muscle-specific MRI grading to ensure reproducibility across institutions will require the development of reference atlases, uniform reporting templates, and targeted radiologist training initiatives. Artificial intelligence and machine learning algorithms hold promise as a means of enhancing the accuracy and consistency of muscle invasion assessment, potentially broadening access to this grading system across centers with varying radiologic expertise.

Despite these limitations, the present findings make several important contributions to NPC prognostication. This study provides the first systematic evidence for muscle-specific grading in the IMRT era, demonstrating robust prognostic value across multiple survival endpoints where traditional staging parameters have failed to discriminate outcomes. The practical nature of the present MRI-based approach, utilizing conventional sequences available in most institutions, offers immediate clinical utility while identifying critical areas for future investigation. The consistency of the results across all four survival endpoints (LFFS, DMFS, PFS, OS) with clear dose-response relationships (mild < moderate < severe) provides compelling evidence for the biological relevance of muscle invasion depth. While prospective validation is essential, the urgent need for refined prognostic tools in the modern treatment era, combined with the clinical practicality of our approach, justifies careful clinical exploration of muscle-specific grading in appropriate institutional settings.

This study’s retrospective, single-center design imposes limitations on the generalizability of its findings. A limitation is the absence of archived per-case adjudication logs in early years, which precluded reporting the requested descriptive counts of consensus cases. Additionally, the moderate invasion subgroup was relatively small (*n* = 76), which may reduce statistical power for some comparisons. Although muscle grading was performed by two blinded radiologists with consensus resolution of disagreements, formal inter-observer agreement statistics (such as kappa coefficients) were not calculated, thereby limiting assessment of the reproducibility of grading.

Heterogeneous induction chemotherapy regimens represent another important limitation, as our analysis treated induction chemotherapy as a binary variable (received vs. not received) without distinguishing between TP, PF, and TPF regimens. Different induction regimens may have variable efficacy profiles, and our analysis cannot distinguish their individual contributions to the observed protective effect. During our study period (2014–2019), institutional protocols evolved, and regimen selection was based on physician preference and patient factors rather than standardized criteria, introducing potential confounding effects.

Additionally, this analysis did not incorporate functional imaging parameters such as diffusion-weighted MRI (DWI) or quantitative PET-based metrics (such as SUV values or metabolic tumor volume), nor did it account for dynamic EBV-DNA kinetics, with these being factors that could have added valuable prognostic insights. The absence of DWI, which is considered the most important sequence for determining the extent of tumor invasion, represents a significant limitation as it may have enhanced the accuracy of muscle invasion grading and contributed to differentiation between tumor invasion and inflammatory changes.

The high rate of missing EBV-DNA data (64.2%) significantly limits the robustness of multivariable modeling. Because EBV DNA availability was limited and non-systematic across the study period (64.2% missing), a meaningful exploratory survival analysis was not feasible; therefore, we report descriptive distributions only and interpret EBV-related findings with caution. Future investigations should prioritize the acquisition of complete molecular datasets to facilitate comprehensive risk stratification. Furthermore, the absence of data on treatment response and patient-reported outcomes prevents a full understanding of how muscle-specific grading impacts therapeutic efficacy and quality of life.

The unexpected finding that the traditional 9th edition staging parameters (T-stage and overall stage) showed no prognostic significance in the present cohort, contrary to most published reports, requires independent validation. This apparent anomaly may reflect the specific characteristics of the present IMRT-treated, endemic region cohort with a high degree of treatment standardization. Nevertheless, generalizability concerns necessitate multi-institutional replication studies.

Prospective, multi-institutional validation studies using comparable imaging protocols, including DWI, standardized chemotherapy regimens, complete molecular profiling, and patient-reported outcome measures, are essential to confirm the clinical utility of muscle-specific grading and establish its role as a complementary tool for risk stratification in routine NPC management.

## Conclusion

These results suggest that the depth of muscular invasion, rather than skull-base involvement, is the predominant anatomical predictor of locoregional control, metastatic spread, and survival in NPC patients treated with IMRT. The muscle-specific MRI grading system described here successfully stratified patients into distinct risk groups across all survival endpoints, in contrast to traditional 9th edition staging parameters, which failed to discriminate outcomes. The sequential, depth-based classification, ranging from superficial (TVP/LVP) to intermediate (prevertebral) to deep (pterygoid/infratemporal) muscular involvement, provides a biologically rational framework that follows natural pathways of anatomical invasion.

The clinical practicality of this approach, utilizing conventional MRI sequences available in most institutions, demonstrates potential for enhanced risk stratification. The substantial risk differences observed between muscle invasion grades (HR 4.4–4.6 across endpoints) suggest potential for risk-adapted treatment strategies, though prospective, multi-institutional validation is required before clinical implementation of such approaches. The protective effect of induction chemotherapy observed for all endpoints further supports the importance of systemic therapy in this disease.

While prospective, multi-institutional validation using standardized protocols is necessary to confirm these findings and establish formal inter-observer reliability, muscle-specific MRI grading can potentially provide added prognostic value beyond conventional skull-base criteria, once validated for clinical use. This approach offers a framework for precision, risk-adapted therapeutic strategies in the modern IMRT era, where traditional anatomical staging boundaries are less effective. Future staging committees may consider incorporating muscle-specific details such as T2 sub-classifications if validated across diverse populations and treatment centers. 

## Supplementary Information

Supplementary Material 1.

## Data Availability

The datasets used and analyzed during the current study are available from the corresponding author on reasonable request.

## Abbreviations

MRI: Magnetic resonance images
STI: Soft tissue involvement
LVP: Levator veli palatini
LP: Lateral pterygoid
EBV: Epstein-Barr virus
ESBI: Extensive skull-base invasion
LSBI: Limited skull-base invasion
IMRT: Intensity-modulated radiotherapy
LFFS: Local failure-free survival
DMFS: Distant metastasis-free survival
PFS: Progression-free survival
OS: Overall survival rates
STI: Soft tissue involvement
HR: Hazard ratio
CI: Confidence interval
