# Supplementary material for: Muscle-specific MRI grading of soft tissue involvement provides additional prognostic value beyond skull base criteria in nasopharyngeal carcinoma: a retrospective study
Source: BMC Cancer. 2025 Nov 12;25:1751. doi: 10.1186/s12885-025-15208-3 (PMC12607154; doi:10.1186/s12885-025-15208-3)
Supplement: Supplementary file 1 — Supplementary Material 1. [file 12885_2025_15208_MOESM1_ESM.pdf]

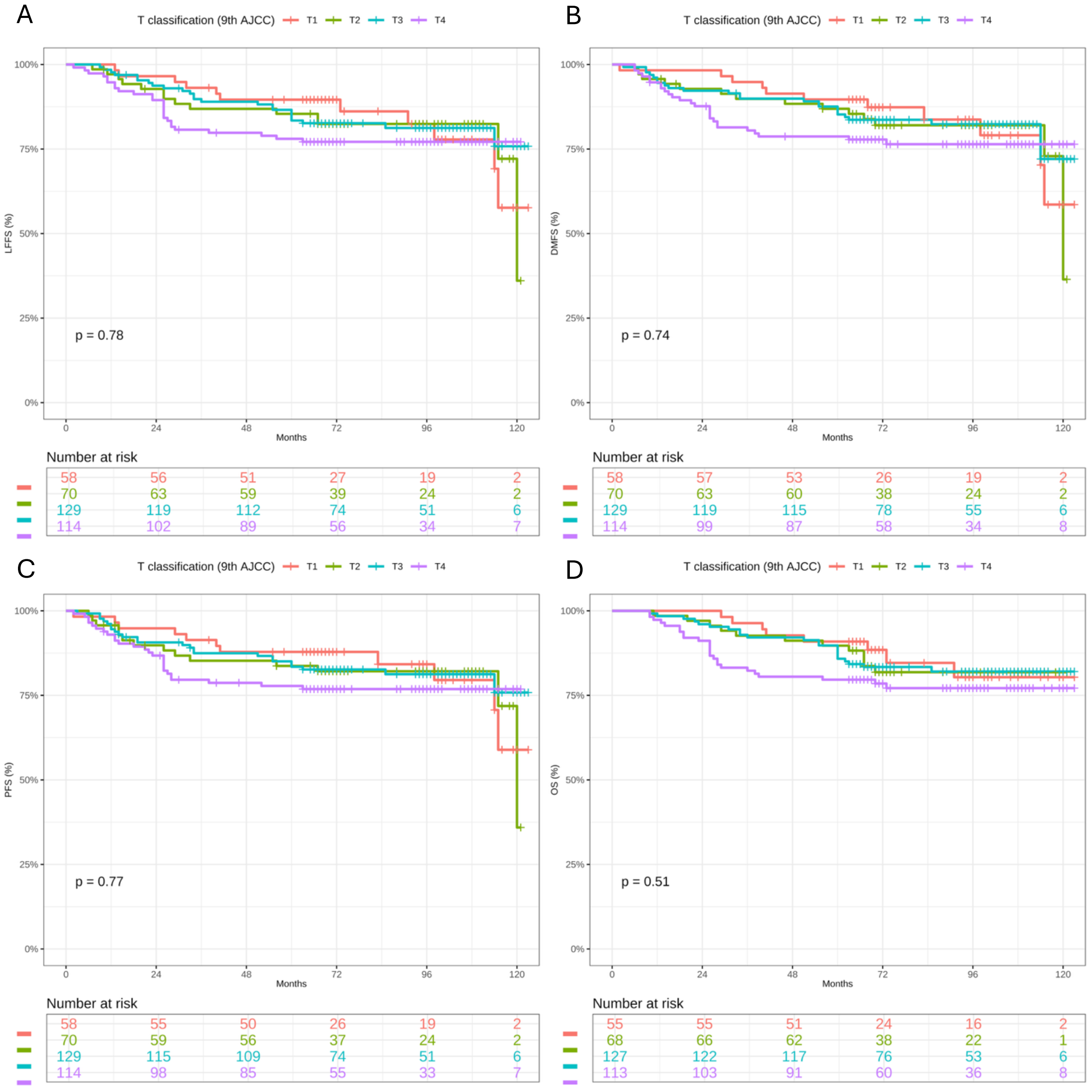

Supplementary Figure S1. Survival analysis by 9th edition AJCC T-stage classification (T1-T4) demonstrates no significant prognostic discrimination across all endpoints: (A) Local failure-free survival (LFFS, P = 0.78), (B) Distant metastasis-free survival (DMFS, P = 0.74), (C) Progression-free survival (PFS, P = 0.77), and (D) Overall survival (OS, P = 0.51).

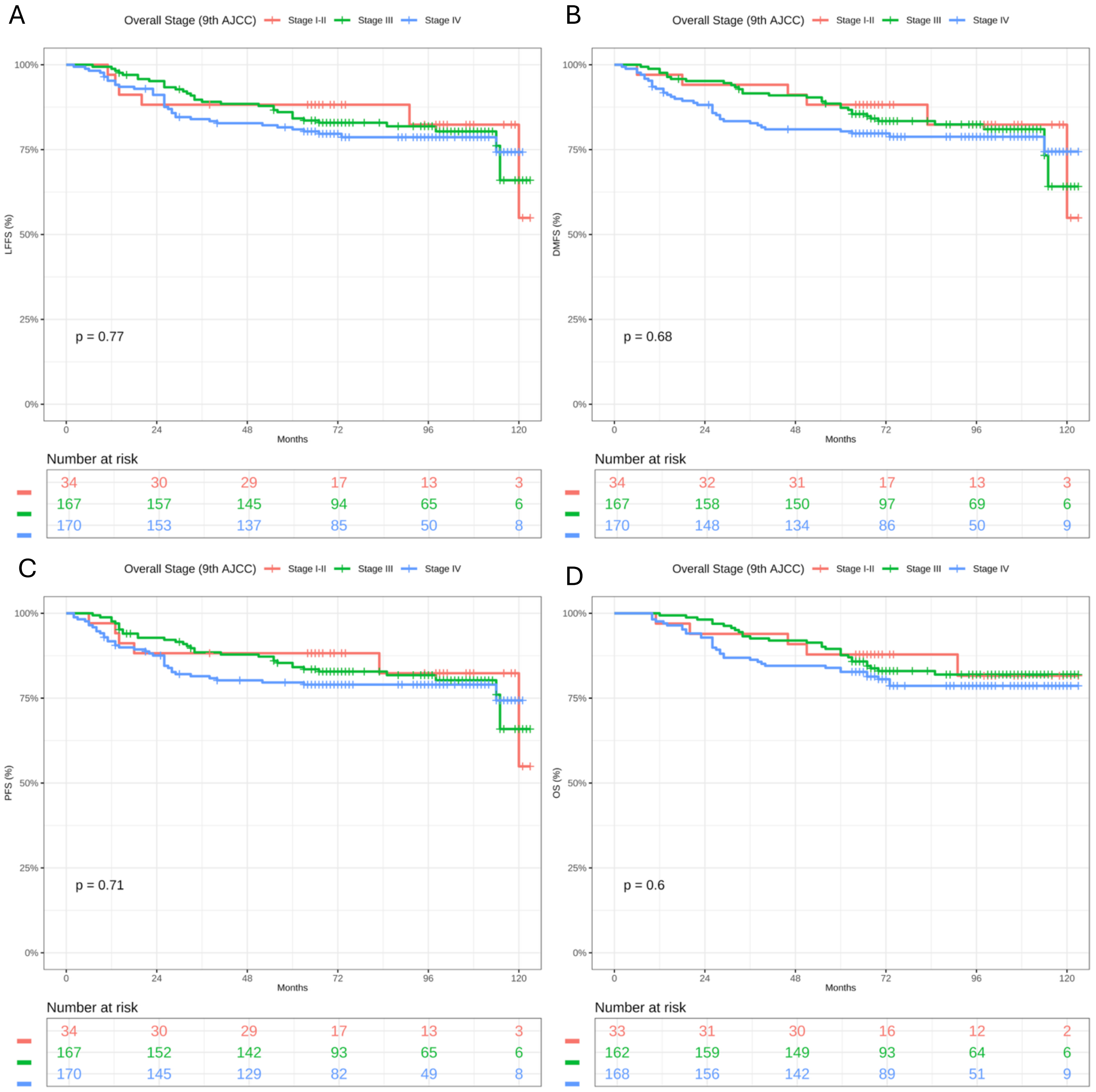

Supplementary Figure S2. Survival analysis by 9th edition AJCC overall stage classification (Stage I-II vs III vs IV) demonstrates no significant prognostic discrimination across all endpoints: (A) Local failure-free survival (LFFS, P = 0.77), (B) Distant metastasis-free survival (DMFS, P = 0.68), (C) Progression-free survival (PFS, P = 0.71), and (D) Overall survival (OS, P = 0.60).
